# The Association of Maternal Height With Mode of Delivery and Fetal Birth Weight at King Abdulaziz University Hospital, Jeddah, Saudi Arabia

**DOI:** 10.7759/cureus.27493

**Published:** 2022-07-30

**Authors:** Shadi M Softa, Nashwa Aldardeir, Faisal s Aloufi, Saad s Alshihabi, Maryam Khouj, Ebtesam Radwan

**Affiliations:** 1 Obstetrics and Gynecology, King Abdulaziz University Faculty of Medicine, Jeddah, SAU; 2 Obstetrics and Gynecology, Urogynecology, King Abdulaziz University Faculty of Medicine, Jeddah, SAU; 3 Medicine, King Abdulaziz University Faculty of Medicine, Jeddah, SAU

**Keywords:** maternal height, maternal and fetal outcome, maternal-child health, gestational hypertension, baby weight, large-for-gestational-age, small for gestational age (sga), emergency obstetric care, c-section, pregnancy surveillance

## Abstract

Study Objectives: The aim of this study was to find if there is an association between maternal height and mode of delivery, as well as an association between maternal height and baby’s weight as a secondary outcome.

Method: This retrospective record review was performed at King Abdulaziz University Hospital (KAUH), Jeddah, Saudi Arabia, including patients admitted between January 2016 to December 2017. All nulligravida with singleton term pregnancies who gave birth were included in this study. Pregnant women with planned elective cesarean section (CS) and incomplete records were excluded. The maternal demographic and clinical data (age, height, weight, hypertension, gestational diabetes (GDM), body mass index (BMI), smoking status, gestational age, regional analgesia during delivery, type of delivery, postpartum hemorrhage (PPH), and episiotomy), neonatal birth weight, and Apgar score were obtained from KAUH computerized records. Our primary outcome was the mode of delivery. The secondary outcome was the classification of neonatal weight into small for gestational age (SGA), appropriate for gestational age (AGA), or large for gestational age (LGA). Maternal height was divided into seven groups. Descriptive statistics using mean and standard deviation were used for continuous variables. Frequencies and percentages were used for categorical variables. Student's t-test and chi-square tests were used to evaluate the differences between continuous and categorical variables.

Result: A total of 1067 women were included in this study. Most were at 40 weeks of gestation age (14.9%) with a mean height of 156.4±6.2 cm. Of the total, 76.9% were spontaneous vaginal delivery without operative assistance, 15.9% were delivered via CS, and 7.2% delivered vaginally with the assistance of forceps or ventouse. The mean neonatal birth weight was 2994 ± 451 gms with most neonates (87.3%) having a birth weight between 2500 and 4000 gms. Most babies were of average weight for their gestational age at delivery. There was a significant negative association between maternal height with CS (p=0.017). Moreover, there was a correlation between maternal height and the baby’s birth weight (p=0.01), and we found that for every 1 cm increase in women’s height, the baby's weight increases by 12.8 gms.

Conclusion: Our study didn’t find an association between maternal height and vaginal delivery or operative vaginal delivery. However, there was an impact of maternal height on CS delivery. Therefore, we suggest screening for short maternal height as they have an increased risk of having an emergency CS. In our secondary outcome, we found a positive association between maternal height and baby's birth weight.

## Introduction

Maternal height is a highly used anthropometric parameter that impacts pregnancy outcomes [1]. Short maternal stature has been related to intrauterine growth restriction, low birth weight, and small for gestational age (SGA) that appears in the ultrasound estimated fetal weight in the third trimester [2-3]. Despite shorter mothers having smaller infants, these mothers often end up with obstructed labor that needs operative assistance and cesarean delivery [4-5]. The Austrian study by Kirchengast and Hartmann found that maternal stature was significantly associated with an increase in Caesarian section (CS) deliveries, which was considerably evident in very short women (<150 cm), who had a very high rate of CS births (> 40%) [5]. A study by Mahmood et al. reported an increased risk of undergoing CS for cephalopelvic disproportion in women shorter than 160 cm. However, 80% of mothers shorter than 160 cm still deliver vaginally [6].

As for the intrauterine growth assessment, it is important for perinatal care to estimate the weight of the fetus and newborn, whether it is SGA, large for gestational age (LGA), or appropriate for gestational age (AGA) [7]. SGA is defined as birth weight less than the 10th percentile, while LGA is a weight that exceeds the 90th percentile of the average weight for gestational age in babies of the same gender. This assessment is crucial as fetuses and newborns exhibit higher rates of mortality and morbidity [8]. Various constitutional factors may affect a child's birth weight, including maternal ethnicity and height [9-10]. Additionally, many risk factors in the antenatal period could be associated with fetal growth, including maternal BMI, gestational weight gain, presence of gestational diabetes, parity, infectious disease, and fetal disease [11-12]. Bodyweight charts for gender and gestational age were structured from perinatal surveys based on millions of databases, and it shows the cut-offs for small for gestational age and large for gestational age, which vary between population and ethnic groups [13]. However, globalization and migration lead to an increased number of interethnic families, which hypothesized that ethnic-specific cut-offs could not be used [14] 

There is very little research that has been performed In Saudi Arabia to connect pregnant women's heights with their mode of delivery and their baby's weight. In this study, our primary aim was to detect whether there is an association between maternal height with the mode of delivery, and our secondary aim was to find the effect of maternal's height on a baby's weight.

## Materials and methods

Study design and setting

This retrospective record review study analyzed the details of women who gave birth at King Abdelaziz University Hospital (KAUH), a tertiary hospital in Jeddah, Saudi Arabia, from January 2016 to December 2017, and was conducted under the Obstetrics and Gynecology department. The recorded data were analyzed from December 2021 to March 2022. All nulligravida with singleton term (37 weeks to 41+6 weeks) pregnant women who gave birth and had an antenatal check-up before delivery were included in this study. The exclusion was pregnant women with planned or elective CS and those with incomplete hospital records. 

Sample size and sampling procedure

There was a total of 7486 deliveries between January 2016 and December 2017. Of these, only 1067 were included in the study according to our inclusion and exclusion criteria. 

Data collection instruments

The maternal demographic and clinical data (age, height, weight, hypertension, gestational diabetes (GDM), BMI, smoking status, gestational age, regional analgesia during delivery, type of delivery, postpartum hemorrhage (PPH), and episiotomy), neonatal birth weight, and Apgar score were obtained from KAUH computerized records. The mother's height and weight were taken prepartum in the triage of the delivery department. BMI was calculated by dividing weight by the square of height and was classified according to WHO classification. 

Our primary outcome was the mode of delivery (spontaneous vaginal delivery, operative vaginal delivery, or CS). The secondary outcome was the classification of neonatal weight into SGA, AGA, or LGA by comparing our data to the dataset from INTERGROWTH-21st [15]. We categorize weight above the 90th percentile to be LGA, less than the 10th percentile to be SGA, and in between AGA. Height was divided into seven groups: ≤ 149cm,150cm-154cm, 155cm-159cm, 160cm-164cm, 165cm-169cm, 170cm-174cm, and ≥ 175). Maternal and neonatal outcomes were assessed according to the categories of maternal height.

Data analysis

We used Microsoft Excel 2016 (Microsoft Corporation, Redmond, Washington, United States) for data entry and IBM SPSS Statistics for Windows, Version 21.0 (Released 2012; IBM Corp., Armonk, New York, United States). Descriptive statistics using mean and standard deviation were used for continuous variables. Frequencies and percentages were used for categorical variables. Student's t-test and chi-square tests were used to evaluate the differences between continuous and categorical variables, respectively. A p-value < 0.05 was considered significant. linear regression was used to find the relationship between a dependent and two or more independent variables 

Research ethics

This study was approved by the Research Ethics Committee of KAUH (No: HA-02-J-008).

## Results

A total of 1067 primigravidae with singleton term pregnancies were studied. Most (n=838, 78.5%) were Saudi Arabian, and their average age at the time of admission was 24.7± 4.6. Their anthropometric data are listed in Table 1. The maternal height at the time of delivery ranged from 122 to 189 cm, with a mean of 156.4±6.2 cm. The average maternal weight at the time of admission was 68.6±13.7 kg, with most (n=1039, 97.4%) not being hypertensive or smokers. 

**Table 1:** Participants' anthropometric data (N=1067)

Data category and range		n (%)
Age Group (years)	<= 20	177 (16.6)
	21-29	740 (69.4)
	>= 30	150 (14.1)
Total number		1067
Maternal Height (cm)	<=149	112 (10.5)
	150-154	267 (25)
	155-159	344 (32.2)
	160-164	249(23.3)
	165-169	71 (6.7)
	170-174	20 (1.9)
	>=175	4 (0.4)
Total number		1067
BMI classification (kg/m2)	<18.5	13 (1.2)
	18.5-24.9	313 (29.3)
	25-29.9	404 (37.9)
	>=30	337 (31.6)
Total number		1067
Gestational age (weeks)	37	86 (8.1)
	38	165 (15.5)
	39	258 (24.2)
	40	277 (26)
	41	144 (13.5)
	Missing	137 (12.8)
Total number		1067
Hypertension	no	1039 (97.4)
	yes	28 (2.6)
Total number		1067
Smoker	no	1057 (99.1)
	yes	10 (0.9)
Total number		1067
Gestational diabetes	no	1008 (94.5)
	yes	59 (5.5)
Total number		1067

All delivery data according to maternal height are listed in Table 2. All pregnancies were carried to term, with the most common gestational age being exactly at the 40 weeks mark (14.9%). A total of 820 women (76.9%) delivered vaginally without operative assistance, only 170 (15.9%) delivered via CS, and 77 women (7.2%) delivered vaginally with the assistance of forceps or ventouse. Of those who delivered vaginally, most (n=826, 77.4%) required episiotomies to complete their deliveries. After delivery, the average neonatal birth weight was 2994 ± 451 gms, with most neonates (87.3%) having a birth weight between 2500 and 4000 gms. Most babies were of average weight for their gestational age at delivery (n=694, 65%). Furthermore, most newborns (77.4%) had an APGAR score of 9 at one minute, getting a full score of 10 at five minutes.

**Table 2:** Delivery data according to maternal height AGA: appropriate for gestational age; SGA: small for gestational age; LGA: large for gestational age

		Maternal Height (cm)						
		<=149	150-154	155-159	160-164	165-169	170-174	>=175
Mode of Delivery	C-section	28	33	59	36	11	1	2
		25.00%	12.40%	17.20%	14.60%	15.70%	5.00%	50.00%
	Operative vaginal delivery	7	22	27	18	3	0	0
		6.30%	8.20%	7.80%	7.30%	4.30%	0.00%	0.00%
	vaginal delivery	77	212	258	193	56	19	2
		68.80%	79.40%	75.00%	78.10%	80.00%	95.00%	50.00%
Regional analgesia during labor	No	9	29	49	27	6	3	1
		8.00%	10.90%	14.20%	10.90%	8.60%	15.00%	25.00%
	Yes	103	238	295	220	64	17	3
		92.00%	89.10%	85.80%	89.10%	91.40%	85.00%	75.00%
Episiotomy	No	32	47	81	48	16	6	2
		28.80%	17.60%	23.50%	19.60%	22.90%	30.00%	50.00%
	Yes	79	220	263	197	54	14	2
		71.20%	82.40%	76.50%	80.40%	77.10%	70.00%	50.00%
labor induction	No	2	3	10	3	1	0	0
		1.80%	1.10%	2.90%	1.20%	1.40%	0.00%	0.00%
	Yes	110	264	334	244	69	20	4
		98.20%	98.90%	96.80%	98.80%	98.60%	100.00%	100.00%
Neonatal Weight (grams)	<1500	0	1	1	1	0	0	0
		0.00%	0.40%	0.30%	0.40%	0.00%	0.00%	0.00%
	1500-2499	15	44	42	17	3	0	1
		13.40%	16.50%	12.20%	6.90%	4.30%	0.00%	25.00%
	2500-4000	97	219	298	228	65	19	3
		86.60%	82.00%	86.60%	92.30%	92.90%	95.00%	75.00%
	>4000	0	3	3	1	2	1	0
		0.00%	1.10%	0.90%	0.40%	2.90%	5.00%	0.00%
Gestational Age	AGA	70	159	229	173	47	10	3
		62.50%	59.60%	66.60%	70%	67.10%	50%	75%
	SGA	29	78	58	37	8	5	1
		25.90%	29.20%	16.90%	15%	11.40%	25%	25%
	LGA	2	2	5	5	3	2	0
		1.80%	0.70%	1.50%	2%	4.30%	10%	0%
	Missing	11	28	52	32	12	3	0
		9.80%	10.50%	15.10%	13%	17.10%	15%	0%
APGAR score at 1 minute	<7	11	24	25	13	8	0	1
		9.80%	9.00%	7.30%	5.30%	11.40%	0.00%	25.00%
	>=7	101	243	319	234	62	20	3
		90.20%	91.00%	92.70%	94.70%	88.60%	100.00%	75.00%
APGAR score at 5 minutes	<7	2	7	7	3	0	0	0
		1.80%	2.60%	2.00%	1.20%	0.00%	0.00%	0.00%
	>=7	110	260	337	244	70	20	4
		98.20%	97.40%	98.00%	98.80%	100.00%	100.00%	100.00%

**Figure 1:**
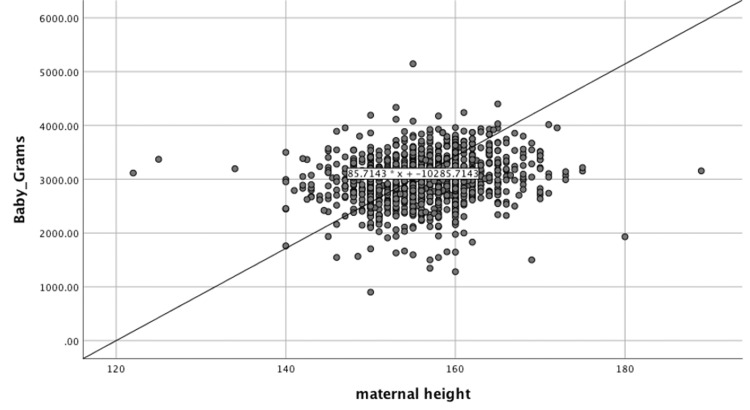
Relationship between maternal height and neonatal birth weight

A chi-square test revealed no significant association between maternal height and vaginal delivery and operative vaginal delivery with a p-value of 0.65 and 0.731, respectively. However, a significant inverse relationship was seen between maternal height and CS delivery (p=0.017). The shorter the mother, the more likely she is to deliver via CS. Linear regression analysis also found an association between maternal height in cm and neonatal birth weight in gms ( p<0.01). For every unit increase in maternal height, an average of 12.8 gms increases in the neonatal birth weight. Figure 1 highlights this relationship.

A chi-squared test also detected an association between the maternal height group and the baby’s weight for gestational age (p=0.00). The taller the mother is, the more likely the chance that she would deliver a baby who is LGA. Also, the percentage of neonates born SGA decreased as the maternal height increased. A significant association (p=0.002) was also determined, between the maternal height and the amount of postpartum bleeding after delivery. The taller the mother was, the less the amount of postpartum bleeding during delivery she went through. No significant relationship, however, was found between maternal height and neonatal outcome, with p-values of 0.223 and 0.757 for an APGAR test at one minute and five minutes after delivery, respectively. Furthermore, no associations between maternal height and the need for episiotomy, the need for labor induction, the presence of gestational diabetes, or the use of regional anesthesia was detected, though a p-value of 0.008 was obtained when associating maternal height with the presence of hypertension. Overall, taller women had a higher percentage of hypertension than shorter ones. A significant association was also concluded between the neonatal birth weight and the mode of delivery (p=0.007), as babies of larger weights were born via CS, while smaller babies were delivered vaginally, and another association was detected between the maternal weight and the neonatal APGAR score at one minute (p=0.003). Women of higher BMIs delivered children with abnormal APGAR scores of less than 7.

## Discussion

In this study, the main aim was to discover if there is an association between maternal height and mode of delivery, with a secondary objective of determining if a mother's height affects her baby's birth weight. We have found no significant relationship between maternal height and delivery either by vaginal or operative vaginal delivery. A significant inverse relationship has been found between maternal height and the occurrence of CS delivery. Moreover, in our sample, shorter women were more prone to have a CS than vaginal or operative vaginal delivery, similar to the results of studies done in both Japan and the United Kingdom [16,17]. This study detected an association between postpartum hemorrhage and the height of the mother; the taller the woman was, the lesser the amount of bleeding she had. However, Japanese and Chinese studies did not find any association between the two factors [16,1]. The use of regional analgesics during delivery was not significantly associated with maternal height in our study, similar to the results of the Japanese study [16]. However, the Chinese paper did find a significant association between the two [1]. Furthermore, there was an association observed between maternal height and hypertension disorders during pregnancy, similar to a Chinese study [18]. In contrast, a study from Japan discovered no association [16].

This study revealed a statistically significant association between maternal height and birth weight, with women of taller heights having children of higher birth weights, similar to several studies performed in Japan, China, and Sweden [16,1,19]. On the other hand, a study by Patra et al. showed opposite results [20]. In our literature, an increase in maternal height by 1 cm is linked to the baby's birth weight increasing by 12.8 gms. This is similar to another study where they found that for every 1 cm increase in maternal height, the baby's weight will increase by 17 gms [21]. In addition, a study by Hugo Azcorra et al. has that found for every 1 cm height increase; there is an increase of 10 gms in baby weight, and a study from Sweden found for every increase of 1 cm in maternal height, there is an increase of 15 gms of baby weight [19,22]. Regarding the baby's birth weight for gestational age classification, we found an association with maternal height. The incidence of SGA increased in shorter mothers, while LGA incidence increased in taller mothers. Likewise, a study from Sweden found similar results [19]. The neonate's outcome of Apgar score at five minutes was insignificant, similar to a Japanese study [16].

We had several limitations in our study that need to be considered. Firstly, the study was performed in a single center; thus, the population is limited and might be biased toward more high-risk patients. The second limitation is that our computerized data were scanned on paper. The third limitation is we had some missing data that could affect our research results.

## Conclusions

Our study didn't find an association between maternal height and mode of delivery. However, there was an impact of maternal height on CS delivery. Therefore, we suggest screening for short maternal height as they have an increased risk of having an emergency CS. In our secondary outcome, we found a positive association between maternal height and a baby's birth weight.

## References

[1] Chan BC, Lao TT (2009). The impact of maternal height on intrapartum operative delivery: a reappraisal. J Obstet Gynaecol Res.

[2] Goldenberg RL, Cliver SP, Mulvihill FX, Hickey CA, Hoffman HJ, Klerman LV, Johnson MJ (1996). Medical, psychosocial, and behavioral risk factors do not explain the increased risk for low birth weight among black women. Am J Obstet Gynecol.

[3] Mongelli M, Gardosi J (1995). Longitudinal study of fetal growth in subgroups of a low-risk population. Ultrasound Obstet Gynecol.

[4] Arulkumaran S, Gibb DM, TambyRaja RL, Heng SH, Ratnam SS (1985). Failed induction of labour. Aust N Z J Obstet Gynaecol.

[5] Kirchengast S, Hartmann B (2007). Short stature is associated with an increased risk of Caesarean deliveries in low risk population. Acta Medica Litu.

[6] Mahmood TA, Campbell DM, Wilson AW (1988). Maternal height, shoe size, and outcome of labour in white primigravidas: a prospective anthropometric study. BMJ.

[7] (2006). WHO Child Growth Standards based on length/height, weight and age. Acta Paediatr Suppl.

[8] Norris T, Johnson W, Farrar D, Tuffnell D, Wright J, Cameron N (2015). Small-for-gestational age and large-for-gestational age thresholds to predict infants at risk of adverse delivery and neonatal outcomes: are current charts adequate? An observational study from the Born in Bradford cohort. BMJ Open.

[9] Ay L, Kruithof CJ, Bakker R (2009). Maternal anthropometrics are associated with fetal size in different periods of pregnancy and at birth. The Generation R Study. BJOG.

[10] Trojner Bregar A, Blickstein I, Steblovnik L, Verdenik I, Lucovnik M, Tul N (2016). Do tall women beget larger babies?. J Matern Fetal Neonatal Med.

[11] Jolly MC, Sebire NJ, Harris JP, Regan L, Robinson S (2003). Risk factors for macrosomia and its clinical consequences: a study of 350,311 pregnancies. Eur J Obstet Gynecol Reprod Biol.

[12] McCowan L, Horgan RP (2009). Risk factors for small for gestational age infants. Best Pract Res Clin Obstet Gynaecol.

[13] Chiossi G, Pedroza C, Costantine MM, Truong VT, Gargano G, Saade GR (2017). Customized vs population-based growth charts to identify neonates at risk of adverse outcome: systematic review and Bayesian meta-analysis of observational studies. Ultrasound Obstet Gynecol.

[14] Zeitlin J, Bonamy AE, Piedvache A (2017). Variation in term birthweight across European countries affects the prevalence of small for gestational age among very preterm infants. Acta Paediatr.

[15] Villar J, Ismail LC, Victora CG (2014). International standards for newborn weight, length, and head circumference by gestational age and sex: the Newborn Cross-Sectional Study of the INTERGROWTH-21st Project. Lancet.

[16] Kuritani Y, Hayashi S, Yamamoto R, Mitsuda N, Ishii K (2020). Association between maternal height and mode of delivery in nulliparous Japanese women. J Obstet Gynaecol Res.

[17] Prasad M, Al-Taher H (2002). Maternal height and labour outcome. J Obstet Gynaecol.

[18] Lao TT, Hui AS, Sahota DS, Leung TY (2019). Maternal height and risk of hypertensive disorders in pregnancy. J Matern Fetal Neonatal Med.

[19] Mogren I, Lindqvist M, Petersson K, Nilses C, Small R, Granåsen G, Edvardsson K (2018). Maternal height and risk of Caesarean section in singleton births in Sweden-a population-based study using data from the Swedish Pregnancy Register 2011 to 2016. PLoS One.

[20] Patra S, Sarangi GD (2017). Association between maternal anthropometry and birth outcome. J Pediatr Assoc India.

[21] Voigt M, Rochow N, Guthmann F, Hesse V, Schneider KT, Schnabel D (2012). Birth weight percentile values for girls and boys under consideration of maternal height (Article in German). Z Geburtshilfe Neonatol.

[22] Azcorra H, Mendez N (2018). The influence of maternal height on offspring's birth weight in Merida, Mexico. Am J Hum Biol.

